# A content analysis of Clinical and Translational Science Award (CTSA) strategies for communicating about clinical research participation online

**DOI:** 10.1017/cts.2018.2

**Published:** 2018-04-23

**Authors:** Elizabeth Flood-Grady, Samantha R. Paige, Nicki Karimipour, Paul A. Harris, Linda B. Cottler, Janice L. Krieger

**Affiliations:** 1 STEM Translational Communication Center, University of Florida, Gainesville, FL, USA; 2 Clinical and Translational Science Institute, University of Florida, Gainesville, FL, USA; 3 Department of Health Education and Behavior, University of Florida, Gainesville, FL, USA; 4 Clinical and Translational Science Institute, University of Southern California, Los Angeles, CA, USA; 5 Department of Biomedical Informatics, Vanderbilt Institute for Clinical and Translational Research, Vanderbilt University, Nashville, TN, USA; 6 Department of Epidemiology, College of Public Health and Health Professions, College of Medicine, University of Florida, Gainesville, FL, USA; 7 Department of Advertising, Department of Health Outcomes and Policy, College of Journalism and Communications, College of Medicine, University of Florida, Gainesville, FL, USA

**Keywords:** Transactional model of communication, clinical research, recruitment participation, Internet

## Abstract

**Introduction:**

There is a dearth of literature providing guidance on how to effectively communicate about clinical research (CR).

**Methods:**

Using the transactional model of communication, a content analysis of the investigator (n=62) and participant (n=18) Web sites of institutions funded through the National Institutes of Health Clinical and Translational Science Award (CTSA) was conducted to identify their strategies (e.g., messages) for communicating about CR participation.

**Results:**

CTSAs targeted investigators with CR participation content across the main Web sites, although most CTSAs (n=55; 88.7%) also included CR participation content for participants. In total, 18 CTSAs (29%) hosted participant Web sites. Participant sites included 13 message types about CR participation (e.g., registry enrollment) and 5 additional channels (e.g., email, phone number) to communicate about CR. However, many CTSA participant Web sites excluded information explaining the CR process and offered CR content exclusively in English.

**Conclusion:**

CTSAs should identify their target audience and design strategies (e.g., messages, channels) accordingly.

## Introduction

Participation in clinical research (CR) or research involving human participants [1] (CR is used broadly to include medical research, clinical trials and observational studies, clinical studies, research studies, trials, and health research) is critical to advancing modern medicine and is an important step in research translation [1, 2]. Yet, a significant proportion of studies experience difficulties recruiting and enrolling an adequate number of study participants [3]. Less than half of adults in the United States are aware of the purpose and opportunities to participate in clinical research [4]. In addition, a lack of trust and high-risk perceptions of the medical research process can often hinder patients’ enrollment in clinical research [5, 6]. Healthcare providers are significant and credible sources of health information [7], yet they rarely discuss opportunities for clinical and medical research with their patients [8]. In general, the public believes that healthcare providers are responsible for educating prospective participants about CR, especially if they are eligible and if participating would benefit them [8]. When providers fail to discuss these opportunities, patients turn to alternate sources and channels for information about research studies (e.g., the Internet). Despite the growing popularity and use of online health information, the strategies used to communicate and educate the public about clinical research studies have not been examined to date.

The Internet is commonly used to search for health information [9–11], with over half (53%) of adults reportedly learning about studies through the Internet [8]. People generally seek information through a combination of keywords that involve a particular health condition, indicator of geographic proximity, and preferred treatment [12]. Universities and hospitals use search engine optimization tactics when designing Web sites in order to increase the visibility of their organization and to draw attention to actively-recruiting trials at their site [13]. Search engine keyword strategies coupled with systems-based search engine optimization tactics may increase the likelihood that consumers will be directed to the Web sites of large university hospitals and medical centers that host studies, such as institutions funded by a Clinical and Translational Science Award (CTSA). The CTSA program initiative, supported by the National Center for Advancing Translational Science (NCATS) [14], aims to improve the quality and speed of research translation and to reduce clinical barriers [15]. NCATS has prioritized reducing barriers to CR participation by funding universities and medical research centers (i.e., CTSAs) to develop and implement innovative strategies to increase recruitment and enrollment [16], including the use of online-based recruitment strategies.

Many academic medical centers provide information about CR and research participation on their main Web sites, with prospective participants averaging just 2 clicks to reach a webpage with information about CR [17]. However, little is known about the completeness of the information available online about CR. Communication literature identifies several factors that are crucial to developing effective information about CR participation, including message completeness, credibility, accessibility (i.e., language of preference), and channel interactivity.

Message completeness refers to the extent to which the information is adequate and balanced in its presentation [18], and is among the most important factors in decision-making [18, 19]. Message credibility reflects the accuracy of a message [19] and trustworthiness of the source presenting the information [20]. Although medical experts are considered the most credible sources of health information [4], this degree of perceived credibility varies according to patients’ racial/ethnic background [21]. Message accessibility—or the extent to which message content can be understood (i.e., interpreted) by the intended target audience—is also important to developing effective information about CR and is associated with perceptions of health information credibility. For example, individuals who are not comfortable speaking English (e.g., Hispanics/Latinos not born in the United States) are less trusting of online health information (i.e., perceive this information as less accurate and credible) than their counterparts who are comfortable speaking English [21]. This suggests that presenting information about CR online exclusively in English not only preclude access to CR content among non-English speakers, but could have significant negative implications for information credibility among certain dual-language speakers (e.g., individuals who *can* but are not comfortable speaking English). Finally, interactivity reflects the extent to which the channel is responsive to user needs and its ability to foster mutual (reciprocal) dialogue [22]. Including multiple strategies (e.g., email, social media) to communicate and exchange information about CR reflects the core tenet of interactivity as it engenders the opportunity for users to serve as both a source (i.e., sender) and receiver (i.e., audience) of communication [23].

CTSA commitment to clinical translational research collaborations across the Consortia [16] has created opportunities for public participation in CR, and ultimately facilitated greater awareness of research opportunities among individuals living in close proximity [24]. Identifying how CTSAs communicate about CR participation online will provide a better understanding about the types of messages and the information prospective participants receive about CTs from credible sources via their preferred channels. Thus, our goal was to identify how CTSAs communicate about CR participation on their CTSA-sponsored Web sites. Findings of this study will have important implications for providing guidance to CTSAs on how to communicate about CR in order to optimize public awareness and enhance understanding of CR among participants.

## Materials and Methods

### Sample Identification

We used the NCATS main Web site to identify the CTSA program hubs and the links to their associated Web sites. At the time of our data collection and analysis, there were 62 CTSA institutions within the Consortia, of which 18 CTSA hubs hosted corresponding (i.e., CTSA sponsored) participant Web sites (i.e., Web sites targeting prospective participants). The 62 CTSA main Web sites and 18 CTSA participant Web sites served as the units of analysis.

### Data Collection and Preservation

Data collection and preliminary analyses were conducted from August 2016 to November 2016. Data collection on each of the CTSA’s respective main Web sites included a review of all content and subsequent links and landing pages embedded within the CTSA main Web site. For the purposes of this study, Web site content that aimed to enhance understanding of CR, educate individuals about CR participation and opportunities, or recruit participants into studies was considered relevant to the research question and collected as data. Content was included if it was presented (i.e., text) or embedded (i.e., link to a video) on the institution’s CTSA-hosted webpage or if it linked to a CTSA-hosted landing page. Content that directed users to an external webpage or Web site (e.g., Office of Research, CISCRP) not hosted by the institution’s CTSA Web site was excluded. Although we did not code content on external webpages, we did code whether Web sites included links to external webpages. The site search tool was used, when available, to locate information that was not apparent on the CTSA main Web sites. Keywords (i.e., participant, volunteer, patient, human subject, participation, research, clinical research, clinical study, clinical trial participation) were used to identify additional site content relevant to the research question. This data collection resulted in the identification of 18 CTSA Web sites for participants.

We used the procedures described above to collect data on the CTSA participant Web sites. Participant CR Web sites were excluded if they were hosted by an organization other than a CTSA, even if the site included a CTSA logo or described their involvement with the CTSA as “affiliated” (e.g., university cancer center’ participant research Web site, university hospital’s research Web site). Population-specific CTSA participant Web sites (e.g., Children’s Hospital of Los Angeles) were also excluded.

Preliminary analysis was conducted from August 2016 to November 2016. Web sites were reviewed, recorded, and coded from December 2016 to March 2017. Three additional CTSA participant Web sites were added in September 2017 and included in our analyses. The prolonged engagement and consistent data collection and observation were conducted as a validity check to ensure we included the most up-to-date materials in the analyses [25]. We preserved our data by collecting screenshots of each Web site and individual webpages with coded content.

### Theoretical Framework

We engaged inductive and deductive approaches to guide the development of the codebook [26]. Two research team members completed an inductive analysis of the 62 CTSA main Web sites to identify strategies for communicating about CR participation. Specifically, we used the transactional model of communication (TMC) to guide the data collection and analysis. The TMC contextualizes communication as an ongoing process or event [27, 28] and identifies 5 key variables important to communication interactions and to understanding message dissemination. We used the TMC as a theoretical framework and identified the source, target audience, messages, channels, and language surrounding CTSA communication about CR participation. These variables broadly capture the factors reported in the communication literature as being crucial to developing effective content about CR participation (i.e., credibility, completeness, accessibility) and to disseminating CR content to participants (i.e., channel interactivity).

Content obtained from the scoping review of the 62 CTSA main Web sites was used to develop operationalized definitions and examples of the categories of variables in the context of CR participation. A codebook was developed and refined through an iterative process using thematic analysis techniques [29] until saturation was reached. During the scoping review, coders identified 18 CTSA participant Web sites. The Web sites were coded separately using the same theoretical framework.

To satisfy our goal to identify communication about CR in order to provide guidance on how to enhance participant awareness and understanding of CR, we engaged in additional inductive analysis procedures to identify specific message themes and message subcategories, as well as additional channels for communicating about CR participation across participant sites. We used the same review procedures and iterative process to develop codes for the CTSA participant Web sites. Some of these additional codes developed for the participant Web sites were later applied to the CTSA main Web sites. The majority of our coding and analyses focus on the CTSA participant Web sites because systematic evaluation of CTSA resources have focused mainly on information (e.g., resources, services) available to investigators [30–32]. This final codebook was used as a final data collection tool. Table 1 includes the operationalized definitions for the 5 variables, as well as the primary categories and operational definitions identified on the CTSA main and participant Web sites.

**Table 1:** Operational definition of the 5 variables and communication categories

Variables	Operational definition	Category	Operational definition
Source	The institution or organization disseminating (e.g., hosting, presenting) messages and content about CR participation	CTSA	Institutional recipient of the CTSA
Target audience	The intended recipients of the messages and content about CR participation. We used primary and secondary to distinguish between the majority and	Investigators	Individuals who are responsible for conducting or leading the clinical research trials, such as researchers, clinicians, clinician-researchers
	minority audiences on Web sites that included information about CR for both audiences	Participants	Individuals who will participate and enroll in clinical research studies, including patients, volunteers, healthy volunteers, prospective participants, subjects, and research subjects
Message	The content about CR participation, including information presented in text and video formats or embedded as webpage links	CR recruitment FAQ	Information (e.g., studios or webinars on recruiting), resources (e.g., training opportunities, templates, core facilities), funding opportunities (e.g., pilot grants, vouchers), and services (e.g., consultations) for investigators to facilitate recruitment or increase enrollment of prospective participants into studies
		Participant CR FAQ	Messages, resources, and educational materials designed to inform prospective participants about CR or increase participation (e.g., CR study types, how to participate). If multiple formats were present, we coded for the format used to present the information (i.e., text, video, link)
Channel	Platform(s) used to host and disseminate content or communicate about CR participation (i.e., ask	CTSA main Web site	The main, NIH/NCATS CTSA-sponsored Web site identified via the NCATS Web site
	questions, solicit feedback)	CTSA participant Web site	The corresponding CTSA-sponsored Web site for participants with a separate, standalone URL, including StudySearch Web sites and Web site-registry combinations
		Email/web contact	Email account hosted by the university that included the name of the CTSA, research, recruitment, or study advocate in the address. The option to send a message through the Web site channel (e.g., submit a form or send an inquiry directly through the site) to ask questions about CR participation, to seek additional information about studies, to inquire about information on the Web site, or to request consultation/service (for investigators on main Web site) was also included in this category
		Social media	Social or other digital media tools presented on the Web site and represented by icons corresponding to the various channels (e.g., Facebook, Twitter). If the site hosted a social media account but did not include the icon on the Web site, it was not included
		Telephone number	Number listed to contact the CTSA to communicate verbally
		Address	Physical address representing the location of the CTSA and for sending traditional, written messages
		Text message	Option to receive information about research studies via text messaging
Language	The type and range of languages used to present the information (e.g., content) about CR participation on the Web sites	Type	Represented by Albanian, Arabic, Cape Verdean, Chinese, English, French, Greek, Haitian Creole, Italian, Khmer, Korean, Mandarin, Portuguese, Russian, Spanish, Vietnamese

CR, clinical research; CTSA, Clinical and Translational Science Award; NIH, National Institutes of Health; NCATS, National Center for Advancing Translational Science. We used a dichotomous scale to record the target audience of the Web sites and to code if messages and channels were present (1) or not present (0) on the CTSA main and participant Web sites. We did not code for language on the main CTSA Web sites.

### Inter-Coder Reliability

Using the final codebook, coders evaluated the content of the CTSA main and participant Web sites. The first author evaluated and coded each of the 62 CTSA main Web sites and each of the 18 CTSA participant Web sites. To ensure inter-coder reliability, an undergraduate research assistant majoring in communication was trained (coder 3) using the codebook as a data collection tool and validated 38% (n=24) of the CTSA main Web sites and the 18 (100%) CTSA participant Web sites. Cohen’s κ [33, 34], which measures the magnitude of agreement between observers (i.e., coders) was calculated for all variables and used to establish reliability. Coders were highly reliable in their agreement of variables. On the CTSA main Web sites, coders reached substantial agreement for target audience (κ=0.76), substantial to perfect agreement for messages and language (κ=0.87–1.00), and perfect agreement for source and channel (κ=1.00). On the CTSA participant Web sites, coders reached substantial agreement for messages (κ ranged from 0.76 to 1.00) and perfect agreement for source, target audience, channels, and language (κ=1.00). Coders reached 100% coding agreement after discussing discrepant codes [17].

### Data Analysis

Data were exported to the statistical software package SPSS 24.0. We computed frequency statistics and conducted a series of χ^2^ to answer the research question. Qualitative excerpts from the Web sites were included as examples for message themes and categories. Statistical significance was set at *p*<0.05 for each test. The results are organized by the source, target audience, message, channel, and language.

## Results

The CTSA main and participant Web sites were considered the primary channels for communicating about CR. Message types varied by target audience and are discussed in terms of the information presented by CTSAs to investigators and to participants across the CTSA main and participant Web sites.

### Source

TMC states that source credibility is important for effective communication [27] and medical research institutions are perceived as trustworthy sources of online health information [35]. CTSAs established their credibility as the source of information about CR on the 62 main Web sites and associated landing pages by including the National Institutes of Health (NIH)/NCATS branding or language about the CTSA grant. We did not consider CTSAs the source of content about CR that appeared on external webpages or Web sites (e.g., University Office of Research, CISCRP) not hosted by the institution’s CTSA Web site.

A smaller proportion of CTSAs were the source of content about CR participation on sites for participants. CTSAs identified themselves as source of information about CR participation for participants with visuals (e.g., buttons “See our participant website”) and by embedding links to the participant Web sites directly on their main Web sites. Because all CTSAs did not promote participant Web sites on their CTSA main site, we also conducted an Internet search to identify additional CTSA hosted.

CTSAs identified themselves of the source of information about CR participation on 18 participant Web site links and associated landings pages by including the NCATS/CTSA grant funding statement on the Web site and describing their site as “developed by,” “copyrighted by,” “sponsored by,” “powered by,” “customized and supported by,” “maintained by,” or “joined together with” the CTSA. CTSAs were also identified as the source of information about CR participation on Web sites for participants that were “sponsored” or “hosted” by the recipient of the CTSA (e.g., the medical college hosted the Web site and was the recipient of the CTSA award as identified by the NCATS Web site).

### Target Audience

Communication is a multi-way process [27], and thus, can involve the exchange of information among a sender and multiple receivers (i.e., target audiences). CTSAs targeted 2 audiences with information about CR participation, investigators, and participants. We used primary and secondary to distinguish between the majority and minority target audiences on CTSA Web sites that included information about CR for both audiences. Investigators (i.e., individuals responsible for conducting or leading the clinical research trials, such as researchers, clinicians, clinician-researchers) were the primary target audience for content about CR participation on the 62 main Web sites and the secondary audience on 12 (66.7%) of the 18 participant Web sites. Participants (i.e., individuals who would participate and enroll in clinical research studies, including patients, volunteers, healthy volunteers, prospective participants, and research subjects) were the primary target audience for information about CR participation on the 18 CTSA participant Web sites and the secondary audience on 55 (88.7%) of the 62 CTSA main Web sites.

### Communication About CR Participation on CTSA Main Web Sites

#### Main Web Site Messages

CTSAs included 2 broad message categories about CR participation on their CTSA main Web sites, CR recruitment FAQs and participant CR FAQs. CR recruitment FAQs included information (e.g., studios or webinars on recruiting), resources (e.g., training opportunities, templates, core facilities), funding opportunities (e.g., pilot grants, vouchers), and services (e.g., consultations), available to investigators to facilitate recruitment or increase enrollment of participants into studies. Participant CR FAQs included messages, resources, and educational materials designed to inform prospective participants about CR or increase participation (e.g., CR study types, how to participate).

At the time of our analysis, all CTSAs (n=62; 100%) included CR recruitment FAQs for investigators on their main Web sites and the majority (n=55; 88.7%) included at least 1 participant CR FAQ on the CTSA main Web sites, χ^2^ (1, n=62)=37.161, *p*<0.001. CTSAs (n=62; 100%) presented CR recruitment FAQs for investigators in multiple formats (e.g., text, video) on their main Web sites. Over half of the CTSAs (n=41; 66.1%) presented Participant CR FAQs for participants in multiple formats on main Web sites (n=41; 66.1%), χ^2^ (5, n=62)=114.323, *p*<0.001. See Table 2 for the type and frequency of communication strategies on the CTSA main Web sites. Although our analysis focused on information appearing on the Web site channels, all 62 CTSAs included additional channels for interactivity between CTSAs and target audiences.

**Table 2:** Type and frequency of communication strategies on Clinical and Translational Science Award (CTSA) main Web sites (n=62)

Communication strategy	n (%)
Target audience	
Investigators (primary)	62 (100)
Participants (secondary)	55 (88.7)
Messages	
Recruitment FAQ	
Yes	62 (100)
No	0 (0)
Participant CR FAQ	
Yes	55 (88.7)*
No	7 (11.3)
Participant CR FAQ format†	
Text	1 (1.6)
Internal link	1 (1.6)
Video	3 (4.8)
External link	9 (14.5)
Multiple	41 (66.1)*
Channels	
Email/web contact	
Yes	62 (100)
No	0 (0)
Social media	
Yes	46 (74.2)
No	16 (25.8)
CTSA participant Web site	
Yes	18 (29)
No	44 (71)*
Language	
English	55 (100)
Spanish	6 (10.9)
More than 2 languages‡	1 (1.8)

CR, clinical research. *Difference is significant at *p*<0.001.

† Percentage of n values does not equal 100; 7 (11.3%) of CTSA main Web sites did not include a Participant CR FAQ.

‡ One Web site included select content about CR participation in 17 languages.

#### Main Web Site Channels

CTSA main Web sites (n=62) were the primary channels used to communicate information about CR to investigators. At the time of our analysis, all 62 institutions included the option to email or to send a message through the Web site to discuss CR participation and 46 (74.2%) had at least 1 social media account on the Web site. A smaller proportion of CTSAs hosted separate, CTSA participant Web sites (n=18; 29%), though the majority of CTSAs did not host a sponsored Web site for participants (n=44; 71%), χ^2^ (1, n=62)=10.903, *p*<0.001.

#### Main Web Site Language

Several factors can limit exposure to a message or inhibit an individuals’ ability to understand or interpret messages [27]. In the current study, we coded for the type of language in which CR content was presented. Although the language in which CR content is presented could fit within the TMC’s conceptualization of messages or as a characteristic of the target audience, we coded language separately to identify the type and ranges of languages used to present CR content.

Of the 55 CTSAs with information about CR participation available to participants on their main Web sites, each (100%) included content about CR participation in English. On the CR webpages (i.e., landing pages with information about CR hosted by the CTSA main site), 6 (10.9%) presented certain content about CR participation in English and Spanish, and 1 (1.8%) presented content about CR participation in more than 2 languages (e.g., English, Spanish, Arabic, Polish).

### Communication About CR Participation on CTSA Participant Web Sites

#### Participant Web Site Messages

All messages on participant Web sites were broadly considered Participant CR FAQs (e.g., messages, resources, and educational materials designed to inform prospective participants about CR or increase participation). CTSA messages about CR participation on participant Web sites were characterized by 5 major themes, with 13 categories (i.e., types) of messages about CR participation across those themes. Tables 3a and 3b includes the message categories (types), message themes, and examples from CTSA participant Web sites. Table 4 provides the frequencies and percentages of communication strategies on CTSA participant Web sites. Below we provide a description of the message themes and the prevalence message types within themes across CTSA participant Web sites.

**Table 3a:** Operational definitions of message categories on Clinical and Translational Science Award (CTSA) participant Web sites

Message theme	Category	Operational definition
Relevance	Active eligibility determination	A search box or visual representation of studies by topic area (e.g., cancer, depression) or check boxes with information for participants to select from to actively determine their eligibility to participate in studies
	Passive eligibility determination	Text describing who is eligible to participate in a study, including what it means to be a healthy volunteer, and information about individuals traditionally underrepresented in CR (e.g., children, women, minorities)
Credibility	About the Web site (and source)	Information about the institution (CTSA) hosting the Web site (e.g., number of facilities, locations, and trials hosted annually, collaborators), content users could expect to find on the Web site (e.g., listing of recruiting studies), or the process for navigating the Web site
	Participant testimonials	Stories and brief accounts from participants describing their experiences participating in a study, enrolling in a registry, or working with research teams. Stories/testimonials in plain text or video format that were embedded directly on the Web site were included, whereas those linking to separate webpages were excluded
CR process	Participant rights	Ethical procedures that occur before a study to ensure participants’ decisions to enroll and remain in a study are voluntary (e.g., informed consent) and precautions taken during to protect participants’ information or to maintain their privacy and confidentiality
	Understanding CR	Description of CR (e.g., clinical trial vs. observational study), the goal and purpose of CR, types of studies (e.g., survey, intervention) and phases of trials (e.g., Phase 2 vs. Phase 3), or clinical procedures (e.g., standard of care vs. placebo) that occur during or as part of a study
	CR risks	Information that could discourage CR participation, such as the potential risk for harm (e.g., minor, severe, or other adverse outcome), medication side effects, or the potential for a treatment to be ineffective
	Safety	Steps in place to ensure participant’s physical safety before and during the study, including IRB review, trial supervision or what participants should do if they are injured during the study
	Study results	Information on the process or timeline for disseminating study results, including where (e.g., clinicaltrials.gov) and how (e.g., article links directly on the Web site) the public can gain access to findings
Participation	Registry participation	Description of a university research registry, including the type of registry (e.g., disease specific or disease neutral), why and how to enroll, the process for being contacted to participate in a study, and the steps to withdraw from the registry
	Other ways to participate	Additional, non-registry ways to participate in CR, such as information on participating in a biobank, or links to local (e.g., university pediatric registry) or national listings (e.g., clinicaltrials.gov) and registries (e.g., ResearchMatch)
Appeal	Intrinsic	Addressed the internal factors that could influence a participant’s decision to enroll in a study, such as the desire to help one’s self or others, or to advance science and medicine. Messages in this category appeared in plain text and as metaphors (e.g., comparison of research participation to being a hero). Metaphors were included as part of the url (site) address, in the name of the Web site, and used throughout the Web site to explain surrounding the decision to participate in research
	Extrinsic	Addressed the external and temporal factors that could affect an individual’s decision to participate in CR, such as transportation, finances (e.g., compensation, cost to participate), or time (e.g., study or visit length)

CR, clinical research; IRB, Institutional Review Board.

**Table 3b:** Themes and examples of messages on Clinical and Translational Science Award (CTSA) participant Web sites (n=18)

Message theme	Category	Example
Relevance	Active eligibility determination	“Find a study or topic or search by condition.” “Search for research studies using various parameters, such as age or gender.”
	Passive eligibility determination	“Anyone can participate in research studies, including children and dependent individuals.” “Research needs healthy volunteers as well as those with medical conditions.”
Credibility	About the Web site (and source)	“(*Website*) was created through a collaboration of (*several*) universities along with many of the state’s healthcare organizations and providers.” “(*Website*) is an easy-to-use tool to search for and find basic information about studies and clinical trials being conducted at the (*name*) university and (*name*) healthcare.” “(*Website*) connects people with cutting-edge researchers and clinical trials to transform laboratory discoveries into treatment and cures.”
	Participant testimonials	“My experience in 3 clinical trials has been phenomenal. Although not cured, my quality of life for the nearly 4 years since diagnosis has been excellent. The professionalism and compassion of my (physician), the nurses, and staff have been truly exceptional.” “The best part of signing up (*for the study*) was being with my peers who have similar challenges that I have, sharing the same problems that I have. Second, the research can benefit someone else, if not me.”
CR process	Participant rights	“Informed consent is the process of learning the key facts about a clinical trial before you decide whether or not to volunteer.” “Your participation in research is completely voluntary and you can change your mind at any time… Ask the study doctor to explain anything you do not understand, take time to talk about the study with those you trust. You should feel comfortable about your decision…”
	Understanding CR	“The term, “clinical trial” can refer to a general health research study but it often refers to a specific type of study. Usually when people use the term “clinical trial” they are referring to a drug or device study.” “Research studies are done to test whether new products are safe and work against disease.”
	CR risks	“Clinical trials still do carry some inherent risk, as most of the research involves new medical treatments.” “Research may involve different types of risks. For a study that asks you to fill out a survey has only minor risks, such as questions that may make you uneasy. For other studies, such as taking an experimental drug, the risks can be much greater (e.g., having a bad reaction to the drug).”
	Safety	“Typically, a medical doctor leads a clinical trial aided by nurses and other study personnel. The person in charge of a study is called a Principal Investigator (PI)…The PI is also responsible for assuring the safety of the participants.” “All research studies that are associated with our (*website*) are reviewed by an Institutional Review Board (IRB)…The IRB’s primary concern is the safety of the patients involved in a study.”
	Study results	“We encourage all researchers to inform their research participants about the specific study results, as a way to thank you for participating in clinical research.” “Once the study is finished, it takes additional time (usually several months) to analyze the data and write the articles that summarize the results. Many studies that are registered on clinicaltrials.gov include a summary of results within a year of study completion.”
Participation	Registry participation	“Creating a volunteer profile allows you to express interest in study categories, which helps our recruitment team match you to trials for which you may be eligible.” “You can remove yourself from the registry at any time. Follow the opt-out link on Welcome page.”
	Other ways to participate	“Find more ways to get involved with research!” Click (*on link to university biobank or national registry*).” “For other studies that are not associated with our (*name of CTSI*), check out the government listing of clinical trials being conducted nationwide: https://www.clinicaltrials.gov/”
Appeal	Intrinsic	“There are many reasons (*to participate*). Often it is hope. Hope for some benefit for yourself; hope for an effective treatment; and longer and better quality of life.” “Join the conquest; help save a life.” “Participating in research is one of the most powerful things you can do to be part of tomorrow’s healthcare breakthroughs.” “Through research, new discoveries are made possible.”
	Extrinsic	“Some studies offer compensation and some do not. Studies listed on (*website*) will usually show the amount of compensation.” “The duration of a study can vary, from weeks to months or longer. Typically, participants in a study will make periodic visits to clinical site, which could be a hospital or other clinical research center.”

CR, clinical research.

**Table 4:** Frequencies and percentages of communication strategies on Clinical and Translational Science Award (CTSA) Participant Web sites (n=18)

Communication strategy	n (%)
Target audience	
Participants (primary)	18 (100)
Investigators (secondary)	12 (66.7)
Message (*Theme*)	
Active eligibility determination (*Relevance*)	16 (88.9)
Passive eligibility determination (*Relevance*)	11 (61.1)
About the Web site (and source) (*Credibility*)	17 (94.4)
Participant testimonials (*Credibility*)	7 (38.9)
Participant rights (*CR process*)	10 (55.6)
Understanding *CR* (*CR process*)	8 (44.4)
CR risks (*CR process*)	7 (38.9)
Safety (*CR process*)	6 (33.3)
Study results (*CR process*)	3 (16.7)
Search active studies (*Participation*)	16 (88.9)
Other ways to participate (*Participation)*	13 (72.2)
Registry participation (*Participation)*	8 (44.4)
Intrinsic (*Appeal*)	16 (88.9)
Extrinsic (*Appeal)*	7 (38.9)
Channel	
Email/web contact	17 (94.4)
Phone number	14 (77.7)
Social media	9 (50)
Mailing address	9 (50)
Text messaging	1 (5.5)
Language	
English	18 (100)
Spanish	4 (22.2)
Mandarin	1 (5.5)

CR, clinical research. Percentage of n values does not equal 100 as strategies were not included across all Web sites.

#### Messages Themes

Five message themes were identified across CTSA participant Web sites: *relevance*, *credibility*, *CR process*, *participation*, and *appeals*. *Relevance*-themed messages explicated the universal applicability of CR participation to all audiences using interactive and passive information strategies. *Credibility*-themed messages reflected the expertise of the source hosting the CR and Web site content [20] as well as the experiences of those who participated in CR in order to increase trust in the institution and research participation. *CR process*-themed messages informed prospective participants about the CR process and aimed to reduce uncertainty about participation. *Participation*-themed messages offered information about research participation and steps (i.e., how to) to enroll in registries and CR studies. *Appeal*-themed messages addressed the internal (e.g., altruism) and external (e.g., temporal, physical) factors that could affect a participants’ decisions to participate in CR (i.e., their interests, needs, and wants) (see Tables 3a and 3b).

The majority (n=16; 88.9%) of the CTSA participant Web sites included at least 1 message type from 4 or 5 of the 5 message themes, whereas 2 (11%) CTSA participant Web sites included at least 1 message type from 3 of the 5 message themes (*p*<0.001). In other words, CTSA participant Web sites were significantly more likely to include message types from at least 4 themes than they were to include message types from only 2 themes. See Table 5 for total message themes included on CTSA participant Web sites.

**Table 5:** Message themes across Clinical and Translational Science Award (CTSA) participant Web sites (n=18)

	Participant Web site																		
Message theme	1	2	3	4	5	6	7	8	9	10	11	12	13	14	15	16	17	18	n (%)
Relevance	x	x	x	x	x	x	x	x	x	x	x	x	x	x	x	x	x	x	18 (100)
Credibility	x	x	x	x	x	x	x	x	x	x	x	x	x	x	x	x	x	x	18 (100)
CR Process	x	x	x	x	x	x	x	x						x				x	10 (55.6)
Participation	x	x	x	x	x	x	x	x	x	x	x	x	x	x	x	x	x	x	18 (100)
Appeal	x	x	x	x	x	x	x	x	x	x	x	x	x	x			x	x	16 (88.9)
Total	4	4	5	5	5	5	5	5	4	4	4	4	4	5	3	3	4	5	

CR, clinical research. CTSA participant Web sites were assigned a random number between 1 and 18 prior to inclusion in this table. x indicates at least 1 message type from the message theme was included on CTSA participant Web site.

##### Relevance

*Relevance*-themed messages (i.e., active eligibility determination, passive eligibility determination) were included on each of the CTSA participant Web sites (n=18; 100%). Within *relevance*-themed messages (e.g., “*Participants who are healthy are often needed to participate in studies*”), messages used to determine active eligibility (n=16; 88.9%) were included more frequently than messages describing passive eligibility (n=11; 61.1%). Half (n=9; 50%) of the CTSA participant Web sites included both *relevance*-themed message types and half (n=9; 50%) included 1 message type from this theme (*p*>0.05). In other words, CTSAs were equally likely to include 1 *relevance*-themed message as they were to include multiple *relevance*-themed messages on participant Web sites.

##### Credibility

*Credibility*-themed messages (i.e., About the website (and source), participant testimonials) were included on each of the 18 CTSA participant Web sites. Within *credibility*-themed messages, information describing the expertise of the source and Web site hosting the CR (i.e., About the website) were included most frequently on the CTSA participant Web sites (n=17; 94.4%). Participant testimonials (i.e., narratives) or messages describing the experiences of those who participated in CR appeared on less than half of the CTSA participant Web sites (n=7; 38.9%). Six (33.3%) of the CTSA participant Web sites included both *credibility*-themed messages types whereas 12 (66.7%) included only 1 of these message types (*p*>0.16). Among CTSA participant Web sites with only 1 *credibility*-themed message, the majority (n=11; 91.7%) included, About the website whereas 1 Web site included participant testimonials (8.3%) (*p*<0.001). In other words, most CTSAs included only 1 *credibility*-themed message on participant Web sites, the majority of which, excluded participants’ descriptions of their experiences with CR (i.e., testimonials).

##### CR Process

Over half (n=10; 55.6%) of the CTSA participant Web sites included at least 1 *CR process*-themed message (i.e., participant rights, understanding CR, CR risks, safety, study results). Within this message theme, participant rights appeared most frequently (n=10; 55.6%), followed by understanding CR (n=8; 44.4%), CR risks (n=7; 38.8%), safety (n=6; 33.3%), and study results (n=3; 16.7%). Among the 10 CTSA participant Web sites that included messages from this theme, 9 (90%) included 2 or more message types whereas 1 (10%) included only 1 type of message from this theme (*p*<0.01). In other words, among the CTSAs that included *CR process*-themed messages on participant Web sites, the majority included multiple messages explaining the CR process.

##### Participation

*Participation*-themed messages (i.e., registry participation, other ways to participate) were included on 18 (100%) of the CTSA participant Web sites. Within this message theme, messages with information and other ways to participate (e.g., participate in our Biobank) appeared on 13 (72.2%) CTSA participant Web sites whereas registry participation appeared on 10 (55.6%) CTSA participant Web sites. The majority of the CTSA participant Web sites (n=17, 94.4%) included both *participation*-themed message types (i.e., other ways to participate, registry participation), whereas 1 (5.5%) CTSA participant Web site included only 1 of these message (*p*<0.01). Among the 16 CTSAs that included *participation*-themed messages on participant Web sites, 9 (56.2%) included 1 message type whereas 7 (43.8%) included both message types (*p*>0.62). On participant Web sites, CTSAs were equally likely to include 1 *participation*-themed message (i.e., either other ways to participate in CR or registry participation) as they were to include both *participation*-message types.

##### Appeal

The majority of CTSAs (n=16; 88.9%) included either an intrinsic or extrinsic social influence message appeal on participant Web sites. Within this message theme, intrinsic messages (i.e., “*The possibility of finding a cure that could help others is one reason to participate*”; “*Be a hero, participate in research*.”) appeared more frequently (n=16; 88.9%) across CTSA participant Web sites than did extrinsic messages (e.g., compensation, time commitment) (n=7; 38.9%). Overall, 7 (38.9%) CTSA participant Web sites included 1 type of *appeal*-themed messages, 9 (50%) included both message types, and 2 (11%) did not include any *appeal*-themed message types (*p*>0.11). Among the 16 CTSA participant Web sites that included intrinsic and extrinsic message *appeals*, 9 (56.2%) included 1 type and 7 (43.8%) included both message types. Thus, CTSAs were equally likely to engage participants in CR using intrinsic and extrinsic message appeals as they were to engage them using only type of message appeal on participant Web sites.

#### Participant Web Site Channels

Of the 18 CTSAs hosting participant Web sites, each (100%) provided at least 1 additional channel to communicate (i.e., ask questions, provide feedback) about CR participation. Email/web contact was the most frequently included communication channel on participant Web sites, with 94.4% (n=17) of Web sites including this channel and only 5.5% (n=1) excluding this channel (*p*<0.001). Also, only 1 of the 17 CTSA participant Web sites featured the option to receive and send text messages about participating in studies (*p*<0.001). Finally, an approximately equal number of CTSA participant Web sites included social media (*p*>0.05) and a local mailing address (*p*>0.05) to communicate about CR.

#### Main Web Site Language

Of the 18 CTSAs hosting participant Web sites, each (100%) included content about CR participation in English. However, there was a significant difference in the percentage of CTSAs that presented content about CR participation on participant Web sites exclusively in English (n=14; 77.8%), compared with CTSAs that presented content about CR participation in both English and Spanish (n=4; 22.2%) or in English, Spanish, and Mandarin (n=1; 5.5%), χ^2^ (2, n=18)=13.000, *p*<0.001. In other words, the majority of CTSAs included information about CR participation exclusively in English on Web sites designed for participants.

## Discussion

This exploratory study presents the first known evaluation of strategies CTSA engage to communicate about participation in CR on their sponsored Web sites. Findings suggest that CTSAs communicate about CR participation primarily with investigators through their main Web sites, as the vast majority of CTSA hubs did not offer a corresponding participant portals (Web sites) to supplement the main Web site. CTSAs established themselves as credible sources of information about CR. Though CTSAs consider investigators their primary audience, many acknowledge that prospective participants could also search and benefit from information about CR. To directly reach this secondary audience, a smaller proportion of CTSAs provided a participant Web site to target participants with information about CR participation. Participant portals incorporated persuasive communication techniques, which theoretically, have the potential to increase knowledge and participation in CR; however, many excluded content about CR processes and offered CR content in only 1 language. Results of this study have important implications for using the TMC as a framework to optimize CR awareness and to enhance understanding of CR among participants.

### Communicating CR Relevance and CR Opportunities to CTSA Target Audiences

The integration of e-technology into CR activities (e.g., recruitment, participation, interventions) has increased substantially in recent years [36]. CTSAs used their main and participant Web site channels generally to provide information about CR participation to investigators and participants. On main Web sites, CTSAs targeted investigators with an abundance of CR information (e.g., messages, services, and opportunities) to optimize CR recruitment whereas content presented on participant Web sites was intended to educate participants about CR and increase enrollment. Although the majority of information on Web site channels was tailored to primary audiences, most CTSAs acknowledged participants and investigators as secondary audiences on the main and participant sites, respectively. Segmenting investigators and participants as distinct target audiences and creating Web site channels with information tailored to these groups is one way to make communication about CR more effective and efficient [37].

On Web sites targeting participants, CTSAs incorporated passive communication strategies (e.g., paragraph of plain text explaining that healthy volunteers can enroll in research), presumably to highlight the universal relevance of CR to participant audiences. Highlighting the relevance of CR to prospective participants—including information necessary to capture the attention of intended audiences motivates individuals to respond to proposed health behaviors (i.e., participate in a study) [38].

Institutions also included active strategies for participants to search for studies at the CTSA institution. Participants could communicate information about themselves to search for studies (e.g., “search by age or gender, “search our site by topic”) and to enroll in university-based research registries (i.e., by clicking either a link or enrolling through the webpage). Including multiple opportunities to participate implies easy access to participate in CR. By providing participants opportunities to communicate information about themselves in their search for studies, CTSAs acknowledged participants as both a source (i.e., sender) and receiver (i.e., target audience) of communication about CR and embraced the core tenet of interactivity [23].

CTSA-sponsored participant Web sites also included messages appealing to prospective participants’ needs and wants (i.e., intrinsic—such as the desire to help others; extrinsic—compensation, length of the study time commitment), presumably to encourage the decision to enroll in the registry or to participate in a research study. Metaphors and other intrinsic appeals addressing participants’ desire to “help others” or to “advance science” appeared with greater frequently across CTSA participant Web sites than did messages responding to the extrinsic (e.g., compensation) needs and wants of prospective participants. Though commonly used to explain complex, clinical information [39, 40], the efficacy of metaphors to increase CR participation and recruitment has received little attention, and warrants future study. Importantly, integrating these strategies to increase enrollment and responding to the altruistic motivations that drive CR participation could also reduce the potential of exploiting and enrolling individuals who may choose to participate solely for economic reasons (e.g., to receive compensation). In line with the CTSA network’s commitment to community engagement [41], researchers should continue to identify the needs, wants, and motivations of participant populations by health condition [42] and within local communities [43] in order to increase CR participation.

### CTSAs As Credible Sources of Incomplete Information About CR

CTSAs established themselves as credible sources of information about CR across the CTSA main and participant Web sites. For example, CTSAs included the NIH/NCATS branding or language about the grant on each of the 62 main Web sites and 18 Web sites for participants. The majority of CTSAs also included information about the institutions’ experience hosting CR (e.g., “We have conducted over 4000 studies”) on CTSA participant Web sites. Source trustworthiness is an important component of message credibility [20]. Including information about the NIH, the grant sponsor and a reputable source of information [35] about CR as well as messages describing the institution’s experience conducting studies could increase trust in the CTSA and in the information presented about CR among stakeholders seeking assistance with recruitment (investigators) or opportunities to participate in CR (participants).

Few CTSAs, however, included information to increase the trustworthiness of CR to prospective participants or content to explain the CR process on Web sites for participants. Institutions largely omitted testimonials from participants describing their experiences participating in CR. Participant narratives (i.e., testimonials) are useful in explaining health, illness, care, healing, and survival [44]. Accounts from local experts (i.e., lay persons with similar health-related experiences) [45], such as individuals who have participated in CR, also serve as credible sources of health information, particularly among minority groups [46]. Future research should explore how the perceived credibility of the sponsoring institution or national-level registry influences potential participants’ decisions to enroll in research. Studies should also examine using participant testimonials in CR recruitment increases perceptions of research credibility and CR participation among minorities.

Despite the limited number of participant portals, only half included information about the CR process. Messages about the CR process are important to educating individuals on what to expect throughout their participation in research. The inability to distinguish between the different types of CR (e.g., therapy vs. observational) or to understand the processes that take place—before and during CR to minimize potential risks and to ensure participant safety—could increase the risk of therapeutic misconception, limit a participant’s ability to understand the scope of their involvement, and have significant ethical implications for recruitment and informed consent. By failing to include content about the CR process on Web sites designed specifically for participants, CTSAs painted an inadequate, unbalanced, and incomplete portrait of CR participation.

Finally, the vast majority of CTSAs included content about CR exclusively in English on their main Web site CR landing pages and on participant Web sites. Presenting information about CR in 1 language (i.e., exclusively in English) across federally sponsored Web sites is inconsistent with CTSA program initiatives (e.g., community engagement) and the Consortia commitment to improving the health of underserved communities [47]. Moreover, presenting information exclusively in English limits equal access to participation, particularly among traditionally underrepresented groups who already experience increased challenges accessing and interpreting CR information online [48].

### Limitations, Strengths, and Future Research

This study is limited by its content analysis methodology. Collecting cross-sectional data is problematic in the digital era, as online information continues to rapidly change and evolve. To overcome this limitation and provide a high degree of validity throughout the data collection process, we monitored the Web sites over a period of 6 months to collect and update the data. As such, offline information about CR participation was not included in this study. Second, it is unlikely our analysis reflects all online efforts from CTSA organizations surrounding CR participation. For example, CTSAs may have purposefully refrained from building public-facing CTSA participant sponsored Web sites beyond the mandatory requirements set forth by the NIH at the start of program initiative or excluded the CTSA brand from their site if it had little meaning for the population in and around the surrounding area. In addition, because we used the list of CTSA hubs identified on the NCATS Web site as the unit of analysis, participant Web sites from CTSA hubs comprised of more than 1 institution (e.g., award given to group of universities, health systems, or hospitals) were excluded unless the CTSA hub hosted its own participant Web site on behalf of all the institutions included in the award. Thus, although CTSA-sponsored Web sites provided a controlled framework for collecting and analyzing the data, the analysis is limited to programs that included the CTSA brand on their public-facing Web sites.

Next, we did not explore how the information about CR affected participant’s decision to participate in a registry or enroll in a specific study. As a next step, researchers should engage community stakeholders to evaluate the messages identified in the current paper in terms of their overall efficacy (i.e., relevance, clarity) at increasing understanding and participation in CR, test the efficacy of the linguistic strategies (i.e., metaphors) used by CTSAs and message framing—the presentation of positive (benefits) versus negative (CR risks) information about CR—in participant recruitment, and to identify participant’s preferences for accessing (e.g., via cell phone or mobile app) and engaging this content. Finally, the results should be interpreted in light of the boundary condition of our conceptualization of CR, which included the multiple types of clinical trials, observational studies, as well as other health and medical research studies involving human participants. Broad use of this term enabled a thorough evaluation of CTSA efforts and strategies for communicating about CR participation online, although the results may not generalize—nor would we expect them to extend to Web sites focusing on 1 type of CR (e.g., phase 1 clinical trial).

### Practical Considerations and Conclusions

Including messages about CR participation on Web sites for participants will not *cause* or *lead* to an increase in CR participation. However, due to the limited understanding of CR and knowledge of opportunities to participate in studies [5], increasing the underlying mechanism of CR recruitment and enrollment in research is in the best interest of researchers and the general population. CTSAs have responded to this call by disseminating information about CR participation to investigators and prospective participants via multiple channels. Organizations should also provide information about CR that explains the process, is accessible to the public, and considers the host of factors (e.g., individual, cultural) that precede CR participation and interplay during decision-making to affect enrollment (e.g., health literacy and information-seeking, racial differences in the decision to enroll in a study). Developing messages that clearly and carefully explain the CR process and that articulate the steps to participate should, theoretically, help to educate prospective participants and reduce barriers to participation.

Based on the findings of our current investigation and our overall experience building public-facing Web sites focused on promoting research awareness and participation, we make the following suggestions to establishing public-facing registries and informational CR Web sites. First, contextualize CR participation as a transactional communication process (i.e., ongoing interaction about CR between unique sources and target audiences) and approach the development and dissemination of CR Web sites and content as 1 feature of this framework. In other words, Web sites (i.e., Web site hosts) and other sources of information about CR participation should reconsider the “if we build it then they will come,” mentality when communicating about CR participation. Rather than expecting consistency in perceptions of information and interpretation across cohorts, CR resources should be developed to strategically engage and communicate *with* potential participants, and not simply to Web site viewers.

For example, the belief among certain minority cohorts that research benefits Caucasians [49] could affect their decision to participate in CR, particularly if the information presented in recruitment excludes the community benefits associated with minority participation (e.g., “Participating in a study could improve the health of your community”). Addressing disparate beliefs about CR in recruitment materials and including information and resources (e.g., Option to email or chat with someone about CR) responding to these beliefs (e.g., acknowledging them, providing alterative information) across channels (e.g., print materials, Web sites, social media) acknowledges that participants’ will approach CR participation in light of their experiences and embraces interactive role in this process.

Second, include relevant stakeholders (e.g., participants, system development teams) in the original design of CR Web site features and content to ensure stakeholder needs are identified early on and managed throughout this process. For example, because certain participant cohorts (i.e., rural adults and adults with low health literacy) misinterpret the common metaphor used to explain randomization (i.e., “randomization is like flipping a coin,” is interpreted as akin to “gambling with one’s health”) [40], affecting their ability to comprehend what it means for treatment [39], including participant representatives in the design of CR content is critical and could lead to identifying additional clinical terms in need of translation. Employing specialists with the appropriate training (e.g., specialization in health or translational communication) to work with medical professionals to translate (i.e., make accessible, easy to understand) certain CR content without compromising the accuracy of the information could also enhance participant stakeholders’ experiences. Communicating regularly with team members involved in the Web site design process (e.g., application developers, interface design specialists) and appointing a central person with the knowledge and skills to intersect with various Web site stakeholder groups is important to sustaining the development process and to producing an effective end product [50]. The collective approach to integrating stakeholder perspectives when designing CR content and Web sites and seeking feedback from relevant stakeholders will be more time effective than disseminating CR content that may or may not resonate with participants or asking system development teams to continually update Web site platforms.

In conclusion, we posit that CTSAs should consider approaching CR participation as a transactional communication process. Under this proposed framework, we offer CTSAs with a template for identifying their target audience and designing strategies (e.g., messages, channels) within Web sites to interact and engage with potential participants about CR, CR participation, and study enrollment. This paradigm shift speaks to the mission and priority of CTSAs, by facilitating an approach that promotes transdisciplinary collaborations and community engagement in advancing CR participation and the public’s health.
